# The Diagnostic Difficulty of Acute, Disabling Neurology: Varicella-Zoster Virus Polyradiculopathy Presenting as Acute Flaccid Paraparesis in an Elderly Male Patient With a History of Poliomyelitis

**DOI:** 10.7759/cureus.95187

**Published:** 2025-10-22

**Authors:** Mitchell Dennis, Mubbashar Hussain

**Affiliations:** 1 Neurology, The James Cook University Hospital, Middlesbrough, GBR

**Keywords:** acute flaccid paralysis, cauda equine syndrome, cerebrospinal fluid analysis, diagnostic and therapeutic challenge, elderly patient care, leptomeningeal enhancement, neuro-infectious diseases, polyradiculopathy, post-polio syndrome, varicella-zoster virus

## Abstract

We present the case of a 74-year-old man who presented with acute flaccid bilateral lower limb weakness, urinary retention, and neuropathic pain in a patient with longstanding significant leg weakness secondary to poliomyelitis. This required a clear and detailed history and examination, alongside rationalised urgent investigation, to rule out time-critical, reversible, and preventable causes of ongoing damage before deciding on a treatment plan.

Initial clinical assessment prompted evaluation for spinal stroke and compressive myelopathy, which was ruled out with urgent magnetic resonance imaging (MRI) of the whole spine. Cerebrospinal fluid (CSF) analysis revealed lymphocytic pleocytosis and elevated protein. This, in conjunction with significant weight loss, raised concern for the possibility of metastatic malignancy. Subsequent viral polymerase chain reaction (PCR) studies confirmed varicella-zoster virus (VZV) infection in the CSF, for which there exists no current consensus treatment. Contrast MRI findings showed leptomeningeal enhancement around the conus medullaris and cauda equina roots. This, coupled with abnormal nerve conduction studies and electromyography (NCS/EMG), led to a diagnosis of VZV-associated polyradiculopathy. Despite 28 days of intravenous aciclovir and a course of high-dose oral steroids, the patient showed minimal to no motor recovery and remained paralysed, dependent on hoists for transfers. The patient did, however, have improvement in neuropathic pain as a result of oral neuropathic pain agents. VZV infection in the central nervous system can have a disastrous outcome and is increasingly detected. Despite this, there is no consensus on evidence-based treatment; therefore, the duration of therapy still requires defining, and other options beyond aciclovir should be explored. This case underscores the diagnostic complexity of acute lower limb weakness, particularly in the context of pre-existing neurological deficit, how a diagnosis of VZV polyradiculopathy can be reached, and the challenges in differentiating this from other neuroinflammatory or malignant causes, as well as the uncertain prognosis even with prompt antiviral therapy.

## Introduction

Varicella-zoster virus (VZV) is a neurotropic virus. Infection causes two separate diseases with differing presentations and complications. The primary infection with VZV is known as chickenpox and is highly contagious through airborne spread, commonly affecting children in England [1]. It presents with a pre-eruptive stage consisting of abnormal sensation in a dermatomal distribution, followed by an eruptive stage marked by vesicular lesions with an erythematous base, which eventually scab over. These can spread over the whole body after starting on the chest, back, or face. During this infectious process, the VZV establishes itself as a latent infection in the sensory ganglia of spinal and cranial nerves. It is then held dormant by the body’s immune system. 

The secondary infection is known as herpes zoster, which results from reactivation of the virus in the sensory ganglion. Typically, the infection is visible in a characteristic dermatomal pattern as the virus travels down its associated sensory nerve to the skin. This usually arises as immunocompetency declines, such as with age, stress, or comorbidity [2]. Pain is significant and often difficult to manage [3,4]. 

Given that VZV is latent in ganglia throughout the neuraxis, it can present itself anywhere in the body as a complicated infection with widespread neurologic complications. Critically, these neurologic complications can occur in the absence of the characteristic dermatomal rash [4,5], a condition known as zoster sine herpete, which significantly increases diagnostic difficulty. 

Viral lumbosacral radiculitis (Elsberg syndrome) is a recognised mimic of compressive cauda equina syndrome. In these cases, it is usually caused by herpes simplex virus (HSV) 2, but around 7% of cases have been reported to be caused by VZV [6]. The documented outcomes are poor, with irreversible damage leading to disability, chronic pain, and even death [7,8]. Despite the potentially devastating outcomes, there exists no current consensus on treatment. 

## Case presentation

Patient background 

This was the case of a 74-year-old man who presented with a history of acute bilateral lower limb weakness and subsequent falls. His past medical history included childhood poliomyelitis, causing chronic right leg weakness (baseline power 1/5 below the knee) and chronic obstructive pulmonary disease. He reported significant unintentional weight loss (approximately 6 kg) in the last six months. Functionally, he was independent indoors with the use of orthoses, and he relied on a scooter to travel outdoors. He lived alone with no need for support with activities of daily living.

History of presenting complaint 

The patient initially sustained a fall as both of his legs suddenly gave way whilst walking. After support from neighbors, he was able to get up. He was then able to mobilise, but with a degree of ongoing weakness in both legs. The following day, his legs gave way again. This time, there was no recovery, and he was brought into the hospital following a long lie at home. He also reported neuropathic-sounding pain travelling down both legs, a loss of feeling in both legs, and an inability to void urine, which required catheter insertion. He denied trauma preceding the falls and reported no rash. 

On clinical examination, cranial nerves and upper limbs were found to be normal. In the lower limbs, tone was reduced bilaterally with absent reflexes throughout. His pre-existing deficit from polio was a weakness in the right lower limb with power 1/5 MRC grade in all ankle and toe movements. There was now power 4/5 in right hip and knee flexion and extension. The left leg had hip flexion and extension of just 1/5, knee flexion and extension of 3/5, and ankle flexion and extension of 2/5. Sensation was objectively intact initially, but he went on to develop reduced vibration and proprioception bilaterally over the next few days, whilst fine touch, crude touch, and temperature sensation remained unaffected. This did not fit a dermatomal pattern and did not ascend or worsen with time. Allodynia also went on to become a feature in the following days. These examination findings fit clinically with a radiculopathy picture, as described in Table 1. 

**Table 1:** Summary of the differentiating features between myelopathy, radiculopathy, and neuropathy

Condition	Affected area	Key symptoms	Reflex changes
Myelopathy	Spinal cord	Weakness, numbness, stiffness, incoordination	Hyperreflexia
Radiculopathy	Spinal nerve root	Weakness, numbness, radiating pain along the nerve	Decreased/areflexia
Neuropathy	Peripheral nerve	Weakness, numbness	Decreased/areflexia

He was unable to mobilise, requiring a hoist for transfers. There was no new bowel incontinence, no other signs of systemic illness, and no rash. Throughout the disease course, neuropathic pain and constipation became complex issues to try to manage, as discussed below. 

Investigations

On admission, the acute onset of flaccid paralysis warranted an MRI of the entire spine with axial images to rule out a spinal stroke and compressive cauda equina syndrome. This revealed no acute abnormalities, with no evidence of spinal stroke and no myelopathic or significant compressive changes. 

Routine blood tests found no contributing abnormalities, and an infectious and autoimmune screen all came back negative. Given the absence of a clear diagnosis on MRI, a lumbar puncture (LP) was then done. Cerebrospinal fluid (CSF) showed markedly raised protein (3.36 g/L), with a lymphocytic pleocytosis (244 x 10^6^, 82% lymphocytes), mildly low glucose (2.9 mmol/L), and raised lactate (4.4 mmol/L). Reference ranges for the normal CSF constituents can be seen in Table 2.

**Table 2:** Normal reference ranges for major CSF parameters. Glucose should be interpreted in relation to serum levels CSF: cerebrospinal fluid; LP: lumbar puncture

CSF constituent	Patient result (initial LP)	Normal reference range [9]
Protein	3.36 g/L	0.15-0.45 g/L
White cell count	244 × 10⁶/L (82% lymphocytes)	<5 × 10⁶/L
Glucose	2.9 mmol/L (serum 4.7 mmol/L)	≥60% of serum glucose (≈2.5-4.5 mmol/L)
Lactate	4.4 mmol/L	1.2-2.1 mmol/L

This, coupled with the clinical syndrome and significant weight loss, raised significant concern that an infiltrative malignancy could be compromising the blood-brain barrier. A computed tomography (CT) scan was done of the thorax, abdomen, and pelvis, which showed no abnormalities or visible signs of lymphoma. 

**Figure 1:**
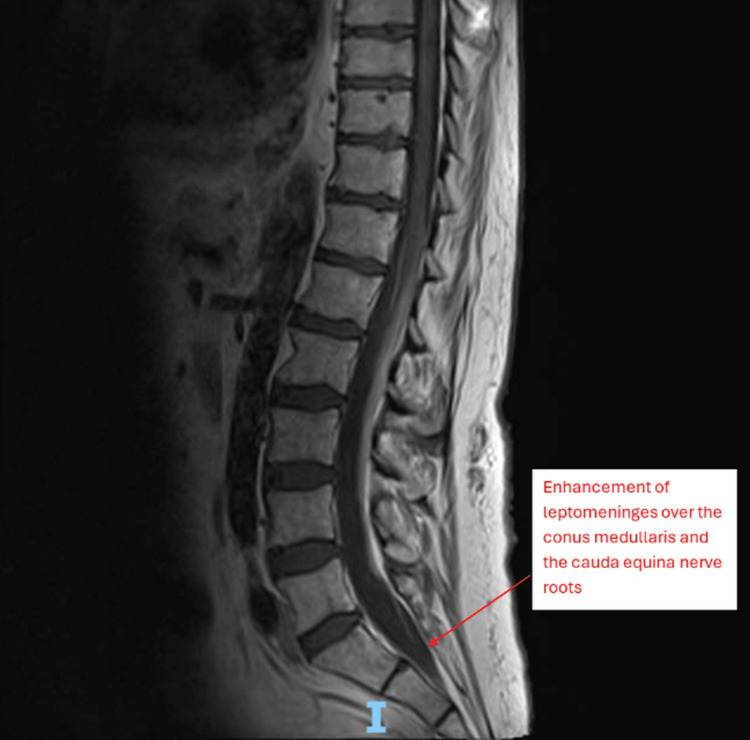
T1 sequence MRI of thoracic and lumbar spine showing leptomeningeal enhancement over the conus medullaris and the cauda equina nerve roots MRI: magnetic resonance imaging

A repeat MRI of the whole spine was then done with contrast, which showed enhancement of the leptomeninges over the conus medullaris and the cauda equina nerve roots. This was reported as highly suspicious for metastatic disease (Figure 1). 

A large volume LP was therefore repeated in order to assess cytology and flow cytometry. Whilst awaiting the results of this, the virology department contacted us to inform us that the initial CSF sample was positive for VZV, shifting the working diagnosis towards an infective polyradiculopathy. With this additional clinical information, the MRI changes could also be explained by an infective process, but malignancy remained a pertinent differential diagnosis. This was discussed with the infectious disease specialist, who agreed it was prudent to treat VZV to limit the replication of the virus in the CSF, but they were not entirely clear whether this was causative or correlative. The infectious disease specialist explained there is no evidence-based standard of treatment, and so they suggested using the same dosing regimen of aciclovir as is used for HSV: 10 mg/kg intravenously three times per day. Dose duration is less well-specified for VZV as compared to HSV; however, it was deemed reasonable to treat for 14 days and correlate with clinical improvement, with a plan to consider a repeat LP around two weeks post-treatment. 

Nerve conduction studies (NCS) were done, which showed no abnormalities in the upper limbs. The lower limb study showed intact lower limb sensory responses but absent or markedly reduced amplitude motor responses. Electromyography (EMG) showed absent volitional activity throughout, with occasional denervation potentials. This was suggestive of lumbosacral polyradiculopathy, or lumbosacral anterior horn cell pathology. 

To exclude metastatic malignancy as a competing diagnosis, a positron emission tomography (PET)-CT scan was done, which showed no abnormal hypermetabolic areas. An MRI was repeated of the spine, which showed no interval changes. The brain was also included in this MRI scan, which found no abnormalities. 

Serial LPs were then repeated every two weeks to monitor progress throughout treatment. After two weeks of treatment, CSF protein remained raised at 4.78 g/L, and the sample tested as weakly positive for VZV. A final LP was repeated two weeks further down the line. The protein had now fallen to 0.56 g/L, and the sample was now negative for VZV, confirming viral clearance after 28 days of treatment. 

Differential diagnoses 

Several important differential diagnoses were considered throughout the evolving clinical picture. Cauda equina syndrome was considered initially, but this was ruled out following an urgent MRI of the spine, showing no compression of the cord. A spinal stroke was also thought to be a possibility given the sudden onset of bilateral flaccid weakness and areflexia; however, the repeat MRI with axial films showed no evidence to support this, and the CSF findings could not be explained by a vascular event. Post-polio syndrome was another potential cause of the initial clinical picture, but abnormal CSF studies and imaging should not have been acutely abnormal for this to be the cause. Guillain-Barré syndrome was also considered, given the bilateral areflexic flaccid paralysis, but the abnormal white cell count in the CSF made this very unlikely. The CSF findings were supportive of an infiltrative process such as a malignancy, but they could also be explained by an infective or inflammatory process. The degree of leptomeningeal enhancement seen on contrast MRI raised further concern for neoplastic meningitis, but this was excluded following a negative PET-CT scan and the absence of malignant cells on CSF cytology and flow cytometry. Paraneoplastic and autoimmune causes of radiculopathy were also considered, but the extended screen returned negative. Ultimately, the diagnosis of infective polyradiculopathy was made. This was confirmed by a positive CSF polymerase chain reaction (PCR) for VZV. This was supported by the contrast MRI images showing leptomeningeal enhancement of the conus medullaris and cauda equina, and similar imaging findings have been seen elsewhere in Elsberg syndrome [10,11]. The findings on NCS and EMG add further evidence to this diagnosis, showing a pattern in keeping with a lumbosacral radiculopathy. 

Treatment

Treatment consisted of antiviral therapy, steroids, and supportive treatment. The patient received intravenous aciclovir at a dose of 10 mg/kg three times daily, initiated immediately after the positive CSF PCR result for VZV. Management was guided by the virology and infectious disease teams, who were involved throughout the case. An initial 14-day course was administered, after which a repeat LP demonstrated persistent, albeit weak, viral positivity. Consequently, treatment was extended for a further 14 days. The third LP confirmed viral clearance, and therapy was discontinued after a total of 28 days. 

Given the persistently raised CSF protein and a lack of initial response to antivirals alone, oral prednisolone was started on day 14 following a neurology multidisciplinary team consensus, which felt there was likely an ongoing degree of inflammation. This started at 60 mg daily and was weaned until stopped by a rate of 10 mg per week once the CSF protein had normalised. 

In addition to antivirals, several adjuvant therapies were employed. Neuropathic pain was managed with gabapentin (up to 500 mg three times daily) and nortriptyline (up to 30 mg nightly). Melatonin was used at 2 mg once a day to help with associated sleep disturbance. 

The patient also required close attention to bowel management as they quickly became constipated. Despite trials of stimulant and softening laxatives, he ultimately required a short course of daily enemas to maintain regular bowel function, likely due to the flaccid paralysis. Following acute treatment, he was transferred to a neurorehabilitation facility. 

Outcome and follow-up 

Despite achieving viral clearance following a course of antiviral and steroid treatment, the patient experienced no recovery in motor function of the lower limbs. The pain improved significantly over the course of 1-2 months, following the introduction and up-titration of neuropathic pain relief agents. Constipation improved following a combination of stimulant laxatives and regular enemas. After spending three months in a neurorehabilitation unit, he was discharged home with a hoist, a long-term catheter, and a substantial care package. 

The diagnosis of VZV-induced lumbosacral polyradiculopathy was established based on clinical presentation, MRI findings, CSF analysis, NCS/EMG, and exclusion of other aetiologies. Unfortunately, the prognosis remains guarded with a low likelihood of significant motor recovery.

## Discussion

This case illustrates an uncommon but important neurological manifestation of VZV reactivation, presenting with acute bilateral flaccid paralysis, bladder dysfunction, and neuropathic pain, mimicking other spinal pathologies. It underscores a recurring theme in neurology where CNS pathologies present profound diagnostic challenges, often requiring a multifaceted approach despite advanced imaging and laboratory techniques, as seen in other complex cases like challenging hemangioblastomas [12]. 

The absence of vesicular rash or dermatomal pain further complicated the diagnosis. VZV polyradiculopathy is a challenging diagnosis, especially in immunocompetent adults, but it is important not to miss it, and it necessitates prompt treatment. Early MRI with contrast and, if imaging is inconclusive, LP for CSF analysis is crucial. PCR confirmation of VZV can guide early antiviral therapy, though treatment benefit on motor recovery remains uncertain, especially when initiated late or in severe cases. 

It is also surprising to note that this is the second neurotropic virus that they have been affected by. The patient's history of poliomyelitis may have been a contributing factor to the severity of his presentation. Pre-existing damage to the anterior horn cells could potentially create a microenvironment that facilitates more extensive viral damage or impairs neural recovery following a second neurotropic viral insult. The limited clinical response in this patient may reflect irreversible neural injury at presentation or the unique neurotropic behavior of VZV at the root level. Literature on prolonged antiviral treatment post-clearance remains sparse, and management should be individualized. Awareness and early intervention for other symptoms, notably pain and constipation, are key both for patient comfort and potential for therapeutic success [13]. 

## Conclusions

This case underscores that VZV must be included in the differential for acute cauda equina syndrome and Elsberg syndrome-like presentations, even in the absence of a rash. Heightened clinical suspicion leading to early CSF PCR testing and empiric antiviral treatment is key, as motor outcomes can be devastating despite therapy. There is currently limited data on treatment guidance and prognosis in people with this condition. The potential for devastating outcomes, as in this case, warrants further exploration into what could be done to maximise the chance of achieving the best possible outcomes for patients. Early symptom support is paramount, and control of this can be a realistic treatment goal. 
